# Cobalamin Deficiency: Clinical Picture and Radiological Findings

**DOI:** 10.3390/nu5114521

**Published:** 2013-11-15

**Authors:** Chiara Briani, Chiara Dalla Torre, Valentina Citton, Renzo Manara, Sara Pompanin, Gianni Binotto, Fausto Adami

**Affiliations:** 1 Department of Neurosciences, Neurological, Psychiatric, Sensorial, Reconstructive and Rehabilitative Sciences, University of Padova, Via Giustiniani 5, Padova 35128, Italy; E-Mails: chiara.dallatorre83@gmail.com (C.D.T.); sara.pompanin@studenti.unipd.it (S.P.); 2 IRCCS San Camillo, Venezia 30011 Italy; E-Mails: valentinacitton@gmail.com (V.C.); rmanara@unisa.it (R.M.); 3 Neuroradiology, University of Salerno, Baronissi 84081, Italy; 4 Department of Medicine, Hematology Unit, University of Padova, Padova 35128, Italy; E-Mails: gianni.binotto@unipd.it (G.B.); f.adami@unipd.it (F.A.)

**Keywords:** vitamin B12, cobalamin, neuropathy, subacute combined degeneration, neuroimaging

## Abstract

Vitamin B12 deficiency causes a wide range of hematological, gastrointestinal, psychiatric and neurological disorders. Hematological presentation of cobalamin deficiency ranges from the incidental increase of mean corpuscular volume and neutrophil hypersegmentation to symptoms due to severe anemia, such as angor, dyspnea on exertion, fatigue or symptoms related to congestive heart failure, such as ankle edema, orthopnea and nocturia. Neuropsychiatric symptoms may precede hematologic signs and are represented by myelopathy, neuropathy, dementia and, less often, optic nerve atrophy. The spinal cord manifestation, subacute combined degeneration (SCD), is characterized by symmetric dysesthesia, disturbance of position sense and spastic paraparesis or tetraparesis. The most consistent MRI finding is a symmetrical abnormally increased T2 signal intensity confined to posterior or posterior and lateral columns in the cervical and thoracic spinal cord. Isolated peripheral neuropathy is less frequent, but likely overlooked. Vitamin B12 deficiency has been correlated negatively with cognitive functioning in healthy elderly subjects. Symptoms include slow mentation, memory impairment, attention deficits and dementia. Optic neuropathy occurs occasionally in adult patient. It is characterized by symmetric, painless and progressive visual loss. Parenteral replacement therapy should be started soon after the vitamin deficiency has been established.

## 1. Introduction

Vitamin B12 or cobalamin (formerly known as cyanocobalamin, because of an artifactual cyano group added to the cobalamin molecule during the original extraction procedure from the liver) is produced by bacteria in the large bowel of humans and by external bacteria and fungi. However, cobalamin from the former source is not absorbed, and humans need to introduce it solely from the diet [1]. The major sources of cobalamin are animal proteins, mainly meats and eggs [2]. An average non-vegetarian diet in western countries contains 5 to 8 μg of cobalamin per day. The recommended daily allowance is 2.4 μg for men and non-pregnant women, 2.6 for pregnant women, 2.8 μg for lactating women and 1.5–2 μg for children up to 18 years. Since a vegetarian diet supplies no more than 0.5 μg/day of cobalamin, most vegetarians are at risk of cobalamin deficiency [3].

Cobalamin is stable at cooking process temperatures; the likelihood of its transformation into inactive compounds by ascorbic acid [4] has not been later confirmed. Since the total-body cobalamin content is two to 5 mg in adults, a complete discontinuation in the absorption will take 3–5 years to deplete cobalamin stores.

## 2. Vitamin B12: Physiology and Causes of Deficiency

Cobalamin from biological sources is provided in the coenzyme form (5′-adenosylcobalamin) as a protein-vitamin complex, through non-specific protein-vitamin binding. At the low pH of the stomach, proteolytic digestion by pepsin occurs, which is the prerequisite for cobalamin release. Once released, cobalamin rather than the gastric intrinsic factor (IF) binds preferentially to haptocorrin (R-protein), a high-affinity cobalamin-binding protein in the saliva and gastric juice, resulting in the so-called “holo-R-protein”. In the second part of the duodenum, pancreatic proteases degrade the holo-R-protein complex, and the resulting free cobalamin binds to IF. The cobalamin-IF complex is stable and resistant to proteolysis in pH ranges of 3 to 9. Ileal IF-cobalamin receptors are selective for IF-bound cobalamin and not for R-bound cobalamin; thus, ileal pancreatic proteases are necessary as much as gastric peptic proteases to ensure optimal cobalamin absorption [1]. IF secretion parallels the secretion of gastric acid, being stimulated by food and inhibited by H2 blockers, as well as by proton pump inhibitors [5]. Long-term use of these two classes of drugs may lead to food-cobalamin malabsorption.

The final step of cobalamin absorption takes place in the ileum through specific membrane-associated IF-cobalamin receptors on the enterocytes. The ileal IF-cobalamin receptor is composed of a protein complex called “cubam” [6]. Dysfunction of the cubam complex leads to Imerslund-Gräsbeck syndrome. Further steps occurring in the enterocytes are ill-defined. As a matter of fact, three to 5 h after entering the enterocyte, the cobalamin appears in the portal blood bound to transcobalamin II (TC II), a specific protein secreted unidirectionally across the basolateral surface of the enterocyte.

The TC II-cobalamin complex has a very short plasmatic half-life, being rapidly cleared mostly because of its rapid binding to specific TC II surface receptors on several cells [7]. Once internalized by endocytosis, cobalamin is dissociated from TC II, then reduced and converted to coenzyme forms *i.e.*, adenosylcobalamin (coenzyme of methylmalonyl-CoA mutase, which converts methylmalonyl-CoA to succinyl-CoA) and methylcobalamin (a coenzyme of methionine synthetase which catalyzes the transfer of methyl groups to homocysteine to form methionine). In the latter reaction, 5-methyl-tetrahydrofolate donates its methyl group to cobalamin, regenerating methylcobalamin. In its turn, methionine can be adenylated to form adenosyl-methionine, which, in turn, donates its methyl group in a large number of biologic methylations involving proteins, neurotransmitters and nucleic acids.

### 2.1. Cobalamin-Folate Relationships

Tetrahydrofolate (THF) is required for the activity of thymidylate synthetase (TS) and DNA synthesis. Other than folate precursor deficiency itself, also cobalamin deficiency contributes to functional intracellular deficiency of THF. Cobalamin indeed is required by methione synthetase for the generation of methionine from homocysteine; meanwhile, THF is generated from 5-methyl THF. In fact, only THF can ultimately act as a coenzyme of the TS.

As a consequence of the deficient TS activity, the synthesis of deoxythymidine monophosphate (dTMP) and deoxythymidine triphosphate (dTTP) from deoxyuridine monophosphate (dUMP) via thymidylate synthase is impaired and dUMP (and, eventually, deoxyuridine triphosphate (dUTP)) accumulates. Since DNA-polymerase does not distinguish dUTP from dTTP, increased amounts of dUTP are misincorporated into DNA. Appropriate DNA-repair enzymes (DNA uracil glycosylase) recognize the faulty incorporation and excise dUTP, but, lacking an adequate dTTP supply, effective repair does not occur, leading to DNA strands breaking.

Megaloblastic changes due to cobalamin or folate deficiency are clinically indistinguishable. The cause of cobalamin deficiency is not generally revealed until specific laboratory tests are done; on the contrary, the recent patient’s history may give clues to the possible folate deficiency. Likely due to the widespread folate supplementation in Western countries, the hematologic picture of cobalamin deficiency is often attenuated, and neurological presentations may become more common and overlooked. It is currently not generally accepted that folate deficiency may induce neurological manifestations, so that the occurrence of neurological symptoms in the presence of folate deficiency should prompt investigations aimed at ruling out cobalamin deficiency [1].

### 2.2. Causes of Cobalamin Deficiency

A strict vegetarian (vegan) diet contains very little cobalamin; less strict vegetarians (lacto-vegetarians, ovo-vegetarians and lacto-ovo-vegetarians) may have subclinical deficiency, as shown by increased blood methylmalonic acid (MMA) and homocysteine concentrations [8]. Since there is clear evidence of abnormal cobalamin metabolism in vegetarians and hyper-homocysteinemia is a risk factor, especially for stroke and vascular dementia, vegetarians are advised to take cobalamin supplements lifelong [3].

In developed countries, the diet is rich in meat and cobalamin-rich foods; thus, malabsorption is the most common cause of cobalamin deficiency. Infants born to vegetarian mothers are at risk of cobalamin deficiency and may present with megaloblastic anemia, involuntary movements and skin pigmentation [9,10]. Most people with insufficient cobalamin intake are, however, poverty-imposed vegetarians living in developing countries and represent a worldwide health problem.

Some of the subjects with subclinical cobalamin deficiency may have normal serum cobalamin concentrations and may be classified as “normal asymptomatic subjects”, although the minimum concentrations of serum vitamin B12 for optimal neuronal health are still unknown, especially in the later stages of life.

### 2.3. Diseases Leading to Insufficient Cobalamin Absorption

In atrophic gastritis or partial gastrectomy, in patients with a long history of *H. pylori* infection, as well as in patients on long-term anti-acid therapy, insufficient pepsin or gastric secretion and inadequate proteolytic digestion there is a failure to dissociate cobalamin from food, thus preventing its absorption [11,12,13]. Moreover, inadequate functional gastric mucosa, gastrectomy, gastric bypass and atrophic gastritis lead to IF deficiency, which, in turn, causes insufficient cobalamin absorption. However, only 30% of patients undergoing partial gastrectomy will eventually have cobalamin malabsorption, and an even smaller proportion will develop frank clinical manifestation of cobalamin deficiency, such as megaloblastic anemia.

The most frequent cause of cobalamin malabsorption is pernicious anemia [14] in which the atrophy of the gastric parietal cells results in a lack of secretion of both IF and chlorhydric acid. The disease has an incidence of 25/100,000 and affects people aged 60 years or older, although in recent years, there has been an increased number of patients younger than 60. Pernicious anemia is an autoimmune disease sometimes associated with other autoimmune diseases, such as thyroiditis (both Graves and Hashimoto diseases), Addison disease and vitiligo. In pernicious anemia, both anti-gastric parietal cells (precisely, the anti-acid-producing enzyme, H^+^/K^+^ATPase) and anti-IF antibodies can be found. There are two types of anti-IF antibodies. Type I antibodies are specific for the IF cobalamin-binding site; type II antibodies bind to the cobalamin-IF complex, preventing its binding to the specific ileal receptors. Some observations suggest that a different (perhaps cellular) autoimmune mechanism may also be involved. Anti-IF antibodies are important clues to the diagnosis of pernicious anemia, since such antibodies can be found in serum or gastric juice in approximately 60% and 75% of patients with pernicious anemia, respectively. Without the presence of these antibodies, the diagnosis relies on the Schilling test or on “*ex juvantibus*” criterion, *i.e.*, cobalamin supplementation [1].

Lacking or inhibition of pancreatic proteases, such as in severe pancreatic impairment or gastric acid hypersecretion, can result in the inability to degrade the R-protein. This may prevent the transfer of cobalamin from R-protein to IF and, eventually, the interaction of the cobalamin-IF complex with ileal specific receptors.

Diminishing acidity or impairment of mechanical cleansing action in the small bowel may lead to bacterial overgrowth. Many bacteria are greedy of cobalamin and take it actively in the free form, while the absorption of the cobalamin-IF complex is much lower. The fish tapeworm, *Diphyllobothrium latum*, infects humans eating raw or partially cooked fish containing larval forms of the worm. The worm lodge usually in the jejunum, and this position allows it to avidly take up cobalamin for its own growth.

Imerslund-Gräsbeck syndrome is a rare autosomal recessive disorder characterized by genetic defects involving the cobalamin-IF complex receptors with subsequent cobalamin malabsorption and low serum cobalamin concentrations that cannot be corrected by IF administration. The disease appears in the first decade and presents with both hematological and neurological features.

Some drugs, such as H2 antagonists, omeprazole, colchicine, neomycin and biguanides, may reduce cobalamin absorption through different mechanisms: inhibition of IF and acid secretion and transenterocytic transport of cobalamin, respectively.

The most clinically significant among the rare disorders of cobalamin transport is transcobalamin II deficiency, which causes megaloblastic anemia in infancy and is associated with normal cobalamin concentrations. Functionally defective TC II have also been described, but have little, if any, clinical relevance [1].

### 2.4. Clinical Tests to Evaluate Cobalamin Absorption

#### 2.4.1. The Schilling Test

In the Schilling test, radio-labeled cyanocobalamin, *i.e.*, the CN-[^57^Co]cobalamin, is administered orally, and its excretion is measured in the urine within the following 24 h. The test is influenced by a number of factors (including incomplete and/or incorrect collection of urine and renal impairment). Since some of these variables are more easily verified directly with appropriate examinations (folate deficiency, achlorhydria and atrophic gastritis, ileal resection or diseases of ileal mucosa, fish tapeworm infestation) and it is far more practical to supplement B12 deficiency, the Schilling test has not gained widespread use in the clinical setting.

#### 2.4.2. Study of Gastric Functions

In children, assessment of IF and acid content of gastric juice can help differentiate between Imerslund-Gräsbeck syndrome (where both are present), juvenile pernicious anemia (where both are lacking) and congenital IF deficiency (where IF is absent). In adults, gastric acidity upon maximal stimulation helps exclude the diagnosis of pernicious anemia.

### 2.5. Biochemical Indicators of Cobalamin Deficiency

Early clinically markers of cobalamin deficiency, occurring even in the presence of normal serum cobalamin concentrations, are increased serum MMA and homocysteine concentrations. Elevation of both markers distinguishes cobalamin-deficient from folate-deficient patients, most of whom display normal MMA values. However, since a number of variables (*i.e.*, renal impairment, age) can result in false positive elevated serum MMA and homocysteine concentrations, only the reduction in the concentration of these metabolites upon cobalamin supplementation is an unequivocal proof of true cobalamin deficiency.

Serum cobalamin concentrations are measured through an assay method that relies on the competitive inhibition by serum cobalamin to the binding of cyanocobalamin to IF. Though serum cobalamin is a valuable marker of cobalamin deficiency, a number of neurologic disorders have been attributed to cobalamin deficiency in spite of normal or minimally reduced serum cobalamin concentrations. A number of conditions (including folate deficiency and multiple myeloma) can falsely lower serum cobalamin concentrations; on the other hand, some clinical conditions, such as myeloproliferative diseases, some leukemias and lymphomas and active liver diseases, may give falsely normal serum values and obscure true cobalamin deficiency. As a matter of fact, 99% of the patients with hematological or a neurological picture consistent with cobalamin deficiency display concentrations of serum cobalamin lower than the low normal limit (300 pg/mL); among cobalamin-deficient patients, approximately 5% have normal cobalamin concentrations, and 10% of true cobalamin-deficient adults have serum cobalamin values in the low normal range [1].

In clinical practice, normal concentrations of serum B12 do not exclude the diagnosis of B12 deficiency. The analysis of serum metabolite concentrations, such as MMA and homocysteine, can reveal patients with borderline B12 concentrations (<300 pg/mL) [15]. Concentration of MMA or homocysteine can be corrected after treatment for B12 deficiency unless renal failure or other causes of increased metabolites co-exist [12]. Screening for vitamin B12 deficiency should start from clinical awareness of the population at risk, including elderly persons, vegans, alcoholics, malnourished persons and patients with gastrointestinal diseases, neuropsychiatric symptoms or autoimmune diseases. Common laboratory findings suggestive of cobalamin deficiency include macrocytosis with or without anemia and hypersegmented neutrophils. Special attention should also be given to patients on medications, such as proton pump inhibitors (PPIs), H2-receptor antagonists, metformin, colchicine, cholestyramine and patients chronically on anticonvulsants or antibiotics. An adequate cobalamin supply is suggested by serum B12 concentrations above 350 pg/mL [11,16]. Assessment of MMA in patients whose serum cobalamin concentrations are below 350 pg/mL is strongly recommended [17].

If MMA concentrations are elevated, it is recommended to rule out other possible causes of elevated MMA, including renal insufficiency or intravascular volume depletion. Hyperhomocysteinemia can be more sensitive to cobalamin deficiency, but it may also reflect folate deficiency, whereas elevated MMA has a similar sensitivity, but more specificity for vitamin B12 deficiency. Vitamin B12 deficiency is suspected when serum cobalamin concentrations are low (350 pg/mL) and when both MMA and homocysteinemia are elevated, or when MMA is elevated in the absence of renal disease or volume depletion, or when homocysteinemia is elevated in the absence of folate deficiency. Several conditions can falsely elevate or decrease serum cobalamin concentrations, but a normal MMA and homocysteinemia concentration suggest the absence of vitamin B12 deficiency [18].

Thus, in some instances, the diagnosis of cobalamin deficiency requires a high index of suspicion and related biochemical alterations, such as high concentrations of both MMA and homocysteine. Should these two marker be within normal limits, cobalamin deficiency is virtually excluded.

## 3. The Multifaceted Clinical Presentation of Cobalamin Deficiency

Vitamin B12 deficiency causes a wide range of hematological, gastrointestinal, psychiatric and neurological disorders. Megaloblastic anemia is a common early symptom leading to the diagnosis, although neurological symptoms may occur in the absence of hematological abnormalities.

### 3.1. Biological and Morphological Expressions of Cobalamin Deficiency

The mechanisms whereby cobalamin (and folates) deficiency produces megaloblastic changes are not precisely known. Defective DNA synthesis leads to disparity in nuclear-cytoplasmic asynchrony and cobalamin or folate-deficient cells slowly divide until mature daughter cells die in the marrow or are arrested at various stages of the cell cycle. Although megaloblastic changes are most striking in the bone marrow and peripheral blood, many other proliferating cells (epithelial cells lining the gastrointestinal tract, epithelial cells pertaining to the female genital tract) may exhibit megaloblastic features. For instance, megaloblastosis in rapidly proliferating cells of the gastrointestinal tract causes atrophy of the epithelial cells of the luminal lining. This may lead to defects in secretion of IF, which, in turn, may aggravate the vitamin B12 deficiency.

**Figure 1:**
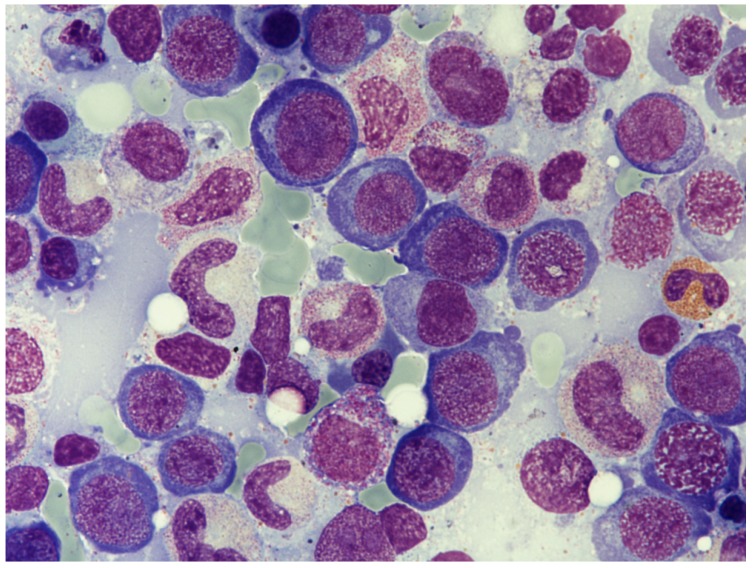
Bone marrow aspiration; May-Grunwald-Giemsa staining; magnification (600×) shows a hypercellular marrow with a reduced myeloid-to-erythroid ratio of about 1:1 (normal 3:1). Megaloblastic basophilic and polychromatophilic erythroblast are prominent, with their reticular and finely dense chromatin. Progenitor myeloid cells also display large dimensions and fine chromatin. Cytoplasmic granulation occurs regularly.

The increase of the mean corpuscular volume is the earliest manifestation of megaloblastosis. Red blood cells appear larger than normal, and some of them may lose the central pale area; furthermore, there may be an abnormal degree of variation in the shape of the erythrocytes in the blood (anisocytosis and poikilocytosis). Red blood cells may show traces of DNA and non-hemoglobin iron (the so-called Howell-Jolly bodies and Cabot rings, respectively). In the bone marrow, ineffective hemopoiesis with numerous mitotic figures and trilinear hypercellularity occurs, mainly of the erythroid series, leading to a reduced myeloid-to-erythroid ratio from 3:1 to 1:1). Megaloblastic changes are more evident at basophilic and polychromatophilic erythroblast stages. Instead of the usual dense chromatin, megaloblastic erythroid progenitors exhibit reticular and finely dense chromatin. In particular, orthochromatic megaloblast retains its immature-appearing nucleus instead of the usual clumped chromatin of the orthochromatic normoblast (Figure 1). In addition, the nucleus may be located eccentrically and may exhibit indentations and or karyorrhexis. Most of the megaloblastic progenitor erythroid cells die in the bone marrow, and macrophages effectively scavenge them, leading to ineffective intramedullary erythropoiesis. Macroovalocytes with incompletely extruded nuclei sometimes circulate in the peripheral blood. Intramedullary hemolysis is easily diagnosed by the mean of elevation of serum lactate dehydrogenase, low reticulocyte count and reduction of serum concentrations of haptoglobin.

Leukopoiesis is abnormal, too. Progenitor cells are increased, display fine chromatin and may reach large dimensions, making it difficult to cross traverse marrow sinuses and to reach peripheral blood. Cytoplasmic granulation occurs regularly; hypersegmented polymorphonuclear neutrophils (PMN) are the hallmark of megaloblastic leukopoiesis in the peripheral blood. For nuclear hypersegmentation to occur, more than 5% of PMN with more than five lobes is required.

Megakaryopoiesis may be involved, with hypersegmented nucleus and liberation of fragments of cytoplasm, generating “giant platelets” in the peripheral blood. However, platelet production and release is impaired, and as a result, various degrees of thrombocytopenia occur.

### 3.2. Hematological Manifestation of Cobalamin Deficiency

Clinical (hematological) presentation of cobalamin deficiency ranges from incidental increased mean corpuscular volume and neutrophils hypersegmentation in otherwise asymptomatic patients to symptoms due to severe anemia, such as angor, dyspnea on exertion, fatigue or symptoms related to congestive heart failure, such as ankle edema, orthopnea and nocturia. Severe anemia may cause dizziness and orthostatic hypotension, too. Food intake may be painful or may be hampered by severe glossitis. Alteration in bowel motility, such as mild diarrhea or constipation, and loss of bladder or bowel control can occur as a result of the involvement of Meissner’s and Auerbach’s plexus within the gastrointestinal tract and the bladder.

The clinical spectrum of cobalamin deficiency has changed significantly over the last few decades. First, it appears that neurological disease is more and more frequent in patients with cobalamin deficiency and mild or absent anemia. Second, only a portion of unequivocally cobalamin-deficient patients display classical abnormal values of hematocrit, mean corpuscular volume and serum lactate dehydrogenase. Furthermore, “standard” morphologic examination of peripheral blood smears fails to recognize the hallmarks of cobalamin deficiency, such as macroovalocytes and hypersegmented neutrophils, since the latter finding is consistently found only in overt cobalamin deficiency. Most of the time, increased red blood cell volume is attributed to other causes, among which are reticulocytosis, liver diseases, chronic alcoholism and myelodysplastic syndromes.

### 3.3. Neurological Manifestation of Cobalamin Deficiency

In the nervous system, vitamin B12 acts as a coenzyme in the methyl malonyl-CoA mutase reaction, which is necessary for myelin synthesis. Vitamin B12 deficiency therefore results in defective myelin synthesis, leading to several central and peripheral nervous system dysfunctions. Regarding the pathophysiological mechanism, lack of adenosylcobalamin (required as a cofactor for the conversion of methylmalonyl-CoA to succinyl-CoA) leads to accumulation of methylmalonyl-CoA, causing a decrease in normal myelin synthesis and incorporation of abnormal fatty acids into neuronal lipids [19].

Neuropsychiatric symptoms may precede hematologic signs and are often the presenting manifestation of cobalamin deficiency. The neurological syndromes associated with vitamin B12 deficiency include myelopathy, neuropathy, neuropsychiatric abnormalities and, less often, optic nerve atrophy.

The spinal cord manifestation, called subacute combined degeneration (SCD) is clinically characterized by symmetric dysesthesia, disturbance of position sense and spastic paraparesis or tetraparesis. The involvement of the posterior and lateral columns of the cervical and upper dorsal parts of the spinal cord is responsible for the impairment of position sense, paraparesis and tetraparesis. The first abnormality is usually sensory impairment, most often presenting as distal and symmetrical paraesthesias at lower limbs frequently associated with ataxia. Almost all patients have loss of vibratory sensation, often associated with diminished proprioception and cutaneous sensation and Romberg sign. Corticospinal tract involvement is common in the more advanced cases, with abnormal reflexes, motor impairment and, ultimately, spastic paraparesis [20]. A minority of patients exhibit mental or psychiatric disturbances or autonomic signs (bladder and erectile dysfunction) [21,22].

Peripheral neuropathy can be seen in 25% of patients with vitamin B12 deficiency [23], and some with acute polyneuropathy have nitrous oxide exposure as the preceding event [15]. Pathologic findings reveal axonal degeneration with or without demyelination. The pathogenic mechanism of cobalamin-deficient neuropathy is a complex network in which also astrocytes and microglia seem to play a role in myelin damage of the type of neuropathy in vitamin B12 deficiency; 76% are axonal, while 24% are demyelinating neuropathy [24]. Some patients may also have subclinical involvement upon electrophysiological testing. Since the frequency of both cryptogenic neuropathy and cobalamin deficiency increases with age, a causal relationship may sometimes be difficult to prove, especially in the elderly, in patients with comorbidities or in chronic treatment with drugs known to cause B12 deficiency, such as the use of metformin in diabetes. An early diagnosis is critical, since the response to treatment depends on the extent of involvement and the timing of replacement therapy [25].

Optic neuropathy due to vitamin B12 deficiency occurs occasionally in adult patients. Lesions in the optic nerve have been demonstrated in post-mortem examination of adults with pernicious anemia. Abnormal visually evoked responses have been reported in patients with pernicious anemia without visual symptoms, suggesting that there may also be subclinical damage to the visual pathway. Optic nerve disease is characterized by symmetric, painless and progressive visual loss. Central and centrocecal scotomas are the main ophthalmologic findings. Clinical response can be seen during the first three months of treatment [23].

Cobalamin deficiency may present with neuropsychiatric syndromes also in the absence of hematological signs. Among the psychiatric presentations mood disorders (both depression and mania), chronic fatigue syndrome and psychosis are notable [26]. Cobalamin deficiency can cause neuropsychiatric symptoms via multiple pathways, including derangements in monoamine neurotransmitter production as cobalamin and folate stimulate tetrahydrobiopterin (BH4) synthesis [27], which is required for monoamine synthesis, and vasculotoxic effects and myelin lesions associated with secondary increases in homocysteine and MMA concentrations [28]. Cobalamin deficiency may also indirectly cause a functional folate deficiency with its secondary metabolic consequences, such as high homocysteine concentrations, decreased monoamine production, decreased *S*-adenosylmethionine (SAM) production and abnormal methylation of phospholipids in neuronal membranes, potentially affecting ion channels and second messengers [27]. In depression, disruption in methylation reactions in the central nervous system necessary for the production of monoamine neurotransmitters, phospholipids and nucleotides [29,30] may be a contributing pathogenic mechanism. Cobalamin is also required for the synthesis of SAM, which is known to have antidepressant properties [31].

Psychosis may be the presenting symptom in vitamin B12 deficiency. Reported symptoms include suspiciousness, persecutory or religious delusions, auditory and visual hallucinations and disorganized thought-processes [27].

An association of vitamin B12 deficiency and depressive symptoms in elderly patients has been documented [32,33]. This finding supports the search for B12 deficiency and eventual replacement of cobalamin in the treatment of depression in clinical practice. A prospective study of outpatients with major depressive disorder reported that adequate concentrations of vitamin B12 correlate with a better response to depression treatment [34].

Symptoms of mania have been described in the presence of vitamin B12 deficiency [35].

Screening for and supplementing with vitamin B12, when appropriate, in the presence of mania is crucial, especially when there is no psychiatric or family history of bipolar disorder.

Vitamin B12 deficiency has been also associated with attention deficits, acute mental-status and acute cognitive changes, with electroencephalography abnormalities [27]. Case reports describe the association of vitamin B12 deficiency and delirium with or without other risk factors, such as dementia and infection [36,37]. Low serum vitamin B12 concentrations have been correlated negatively with cognitive functioning in healthy elderly subjects [38]. The association of vitamin B12 deficiency and cognitive dysfunction has been extensively documented [39], and some authors state that it can be linked to mental decline [40]. Symptoms described include slow mentation, memory impairment, attention deficits and dementia [41]. There is a linear correlation between serum cobalamin concentration and cognitive function, both in healthy elderly people and in patients with Alzheimer’s disease [42]. The available evidence does not allow one to identify a specific profile of cognitive decline, because of the few published cases with complete neuropsychological assessment [43]. In some clinical cases, frontal-dysexecutive syndrome has been documented, occasionally reversible after B12 replacement. Some reports describe patients with preeminent executive impairment with alteration on verbal fluency and response inhibition, together with behavioral abnormalities (mood modification, loss of insight and social awareness, disinhibition and mental rigidity). Authors call frontotemporal dementia-like syndrome the clinical presentation with behavioral disturbances and frontal deficits associated with B12 deficiency [44,45,46]. The possibility of differentiating this cognitive pattern from the common amnesic decline of Alzheimer’s disease through neuropsychological evaluation has been suggested [47].

On a retrospective study on the effects of B12 treatment on neuropsychological function and disease progression in patients with dementia or cognitive impairment, Eastley *et al*. [48] found that vitamin B12 treatment improves cognitive impairment, but does not reverse dementia. Although evidence of vitamin B12 efficacy in improving the cognitive function of people with dementia and B12 serum concentrations is insufficient [49], it is advisable to screen for vitamin B12 in all patients with cognitive impairment [23]. Recently, folic acid and vitamin B supplementation (B12, B6) has been found to slow the atrophy of brain regions that are associated with cognitive decline [50].

### 3.4. Other Effects of Cobalamin Deficiency

Cobalamin deficiency may sometimes result in sterility due to the effects on the gonads; defective bactericidal activity may also be impaired, and increased susceptibility to *Mycobacterium* *tuberculosis* may occur for poorly understood reasons.

## 4. Neuroimaging

Vitamin B12 deficiency may affect both the central (brain, spinal cord and optic nerve) and the peripheral (peripheral nerves) nervous system [51,52].

Since the early 1990s, MRI has been considered pivotal for detecting B12 deficiency-related central nervous system involvement and for excluding possible mimics [53,54]. The main neuroradiologic finding is a typical pattern of myelopathy [55,56,57], though the involvement of neural structures outside the spinal cord has been well documented *in vivo* by MRI.

**Figure 2:**
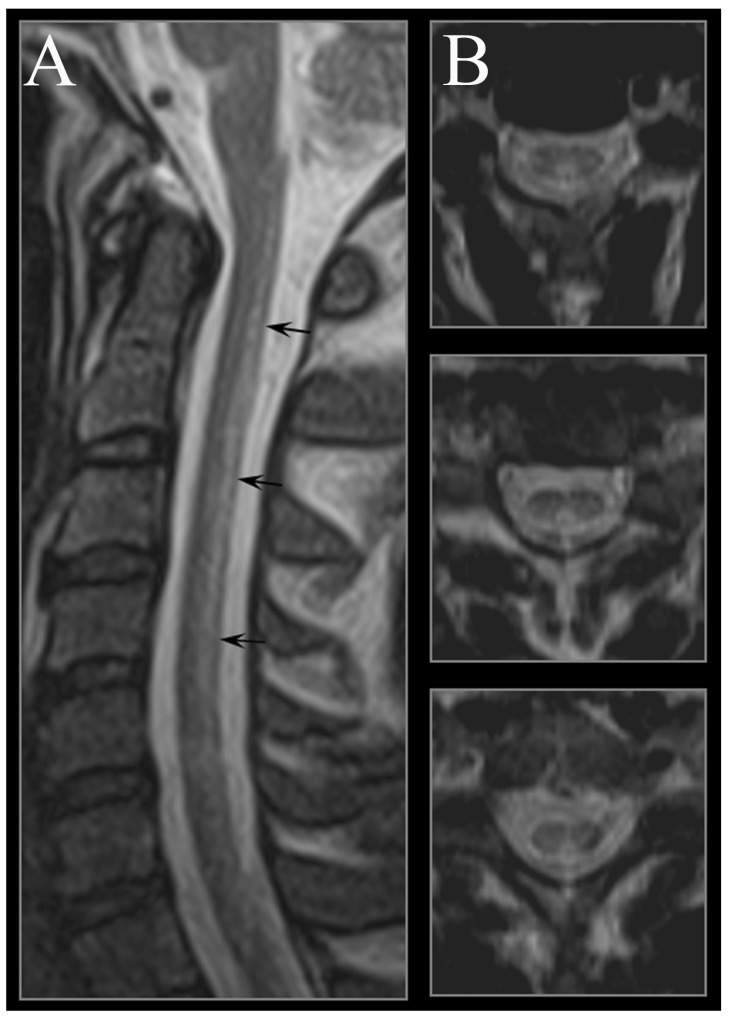
Cervical spinal cord MRI in a 49-year-old male presenting with subacute combined degeneration due to a deficit of B12. (**A**) The midsagittal T2 weighted image shows linear hyperintensity in the posterior portion of the cervical tract of the spinal cord (black arrows). (**B**) Axial T2 weighted images reveal the selective involvement of the posterior columns.

The spinal cord involvement is associated with the most frequent clinical manifestation of vitamin B12 deficiency, namely SCD. The most consistent MRI finding in SCD is a symmetrical abnormally increased T2 signal intensity, commonly confined to posterior or posterior and lateral columns in the cervical and thoracic spinal cord (Figure 2).

In our experience, axial T2 images are more effective in detecting spinal cord lesions, as faint signal abnormalities might be easily overlooked, due to partial voluming on sagittal imaging. In acute and severe cases, the spinal cord might also present as swollen [58]. Involvement of anterior columns has occasionally been reported [59]. T2-hyperintensity of spinal cord columns has been related to demyelination. However, recently, it has been reported on symmetric diffusion weighted imaging hyperintensity in lateral and posterior columns [60] with restricted water diffusion on apparent diffusion coefficient maps, consistent with the co-existence of intramyelin edema. Intramyelin edema in the white matter of the spinal cord is the histopathologic hallmark in experimental models of SCD [61]. Sometimes, enhancement is noted after the administration of gadolinium, due to the disruption of the blood-brain barrier. Spinal MR imaging assists in early diagnosis and treatment of the disease; follow-up MR imaging findings correlate with clinical outcome after treatment with vitamin B12 supplementation. The abnormal MR signals on the spinal cord might either disappear on follow-up after months, or sometimes, it might persist, especially in cases diagnosed and treated at an advanced stage [20,52].

Differential diagnoses of SCD include copper deficiency myelopathy [62], infectious and post-infectious myelitis [20] and multiple sclerosis, even though in the latter condition, lesions are typically asymmetric and do not extend more than 2–3 myelomers.

**Figure 3:**
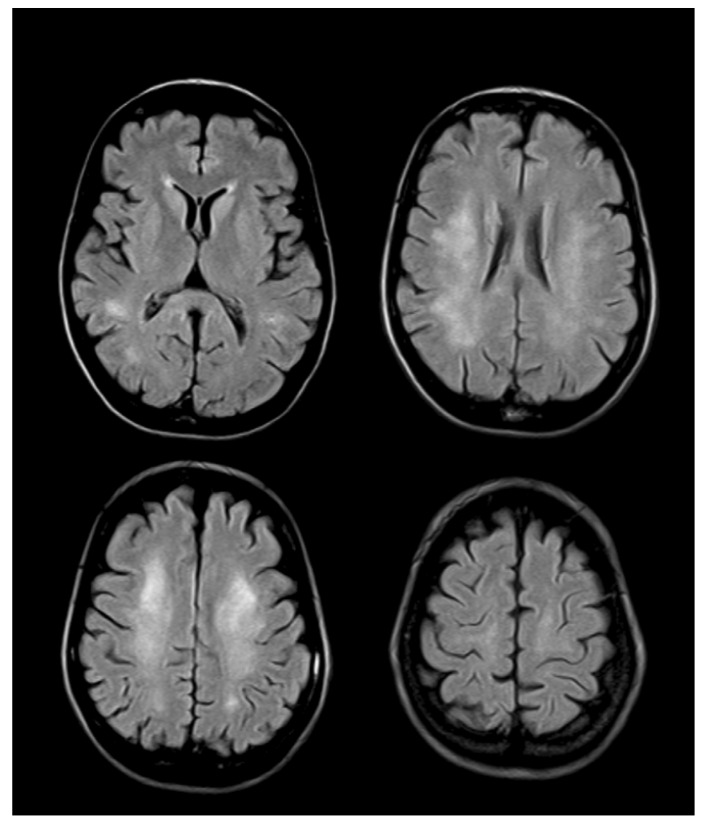
Brain MRI of a 45-year-old female presenting with slowly progressive cognitive impairment. Axial attenuated inversion recovery (FLAIR) images disclose diffuse and symmetric white matter signal hyperintensity.

Brain involvement has been reported in B12 deficiency. Fluid attenuated inversion recovery (FLAIR) and T2-weighted images might demonstrate extensive areas of a high-intensity signal in the periventricular white matter (Figure 3).

In children with inherited cobalamin-related diseases, the damage is limited to the brain and characterized by white matter loss with delayed myelination; MRI has also revealed delayed myelination in infants with nutritional cobalamin deficiency [63]. Recently, a focal signal change has been described both on T2 and DWI in the splenium of the corpus callosum [64]. As folic acid plays an important role in the development of the neural tube, it has also been suggested that vitamin B12 deficit might have some impact on the development of spina bifida and Chiari type 2 malformation [65].

Marchiafava-Bignami disease is a rare disorder causing progressive demyelination and corpus callosum necrosis, primarily associated with chronic alcohol abuse, likely due to vitamin B complex deficiency. Callosal lesions with selective involvement of the middle layers are highly suggestive of Marchiafava-Bignami disease. Concomitant symmetric signal abnormalities might be observed in the centrum semiovale, internal capsule and middle cerebellar peduncle. In some cases, frontal cortex hyperintensity might be detected by FLAIR and DWI imaging (Morel’s laminar sclerosis) [66].

Peripheral nerve abnormalities in B12 deficiency are usually too subtle to be recognized by current ultrasound, CT and MR imaging techniques, while nerve conduction studies reliably unveil the sensori-motor polyneuropathy, due to both demyelination and axonal degeneration. Similarly, neurophysiologic evaluation (and in particular, visual evoked potential) is preferred as a diagnostic tool for optic nerve involvement [67], while neuroimaging has a role in excluding other causes of optic nerve impairment.

## 5. Therapy

Usually, 1 mg/day of cobalamin given intramuscularly for a week, followed by 1 mg/week for four weeks and then 1 mg/month for life, is an appropriate therapeutic regimen. Obviously, therapy should be started after the vitamin deficiency has been established and the causes thoroughly looked for. Oral daily doses of 1–2 mg seem also to be suitable for patients refusing parenteral therapy who have cobalamin malabsorption or pernicious anemia, provided that cobalamin stores have been rapidly supplied by parenteral cobalamin.

Effective therapy reverses megaloblastosis in 24 h and reestablishes normal marrow hematopoiesis in 48 h. The reticulocyte count increases after 3–4 days and peaks after a week; hypersegmented neutrophils remain in the peripheral blood up to two weeks. Rising red blood cell count and hemoglobin will take one week; normalization of the complete blood count requires about eight weeks.

### Neurologic Changes after Effective Therapy

Although most patients respond well to cobalamin treatment, residual neurological abnormalities persist in most. Evidence of improvement or the reversal of neuropsychiatric symptoms varies according to symptom severity, duration and clinical diagnosis. It is commonly accepted that treating deficiencies in the early stages yields better results, as structural and irreversible changes in the brain may occur if left untreated. Vitamin B12 status has been associated with the severity of white-matter lesions, especially periventricular ones, in some [68], but not all, studies [69]. The partial reversal of white-matter lesions has been documented with cobalamin treatment [70], emphasizing the importance of early detection and treatment of vitamin B12 deficiency. A correlation of vitamin B12 treatment and a decrease in MMA and homocysteinemia has been shown [71], suggesting a reversal of metabolic abnormalities. There is evidence suggesting that EEG, visual and somatosensory evoked potentials and P300 latency abnormalities readily improve with treatment, even if no clinical benefits are observed [38].

The follow-up of B12 deficient patients should aim at verifying the compliance to cobalamin supplementation and carefully monitor patients with pernicious anemia, since they are likely to develop iron-deficiency as a result of impaired iron absorption, due to achlorhydria, and are at risk of developing stomach cancer. In some instances, cobalamin supplementation is advocated as a prophylactic measure in gastrectomized patients and in patients undergoing high-flux hemodialysis, as well as in vegetarians or infants born to mothers with pernicious anemia.

## 6. Conclusions

Vitamin B12 is a water soluble substance critical for normal functioning of the nervous system and blood cell formation. Vitamin B12 deficiency may result from pernicious anemia, gastric resection, intestinal malabsorption or a strict vegan diet.

It is often overlooked and may cause several hematological, gastrointestinal, psychiatric and neurological manifestations. Megaloblastic anemia is an early hematological sign, but neurological symptoms may occur also in the absence of hematological abnormalities. SCD, peripheral neuropathy, neuropsychiatric disorders and optic nerve atrophy are the most common neurologic manifestations. The typical MRI finding in SCD is a symmetrical abnormally increased T2 signal intensity, commonly confined to posterior or posterior and lateral columns in the cervical and thoracic spinal cord. Brain involvement has also been reported in B12 deficiency patients with extensive areas of a high-intensity signal in the periventricular white matter.

Physicians should be alert to identifying signs or symptoms of anemia or suspected vitamin deficiency in populations at risk, even in the absence of hematological symptoms or signs. The concentration of serum vitamin B12 is not sufficient for diagnosis, and the metabolites upstream (homocysteine and MMA) should always be looked for. When B12 deficiency is diagnosed, an extensive search of the cause of cobalamin deficiency should always be carried out.

Parenteral replacement therapy should be initiated as soon as possible and associated with a careful, life-long, follow-up of the patients. Early diagnosis is crucial for starting replacement therapy and avoiding irreversible neurological damage.
